# Perceived barriers towards the importance and application of medical research: a source of gender disparity among medical undergraduates

**DOI:** 10.1186/s12909-022-03822-9

**Published:** 2022-11-08

**Authors:** Lina AlQirem, Leen Al-Huneidy, Muhammad Hammouri, Hana Taha, Husam Al-Somadi, Farah Al-Bitar, Razi Kitaneh, Yazan Al-Huneidy, Hussien Al-Somadi, Omar Ashour, Farah Sayed, Dina Mohammed, Raya Abu Tawileh, Abdallah Al-Ani

**Affiliations:** 1 Faculty of medicine, The University of Jordan, Amman, Jordan; 2 Faculty of medicine, The Hashemite University, Zarqa, Jordan; 3 Department of Internal Medicine, Al-Bashir Hospital, Amman, Jordan; 4 Department of Internal Medicine, Al-Shmaisani Hospital, Amman, Jordan; 5 Department of Internal Medicine, Al-Istishari Hospital, Amman, Jordan; 6 Department of Internal Medicine, Arab Medical Center, Amman, Jordan; 7 Office of Scientific Affairs and Research, King Hussein Cancer Center, Amman, Jordan

**Keywords:** Medical education research, Mentoring, Undergraduates, Medicine

## Abstract

**Background:**

Little is known about gender disparity among medical undergraduates in the developing world. Therefore, this study aims to explore the attitudes and perceived barriers among Jordanian medical students, particularly women.

**Methods:**

An online, self-administered questionnaire, developed after an extensive literature review, was disseminated across all six Jordanian medical schools targeting more than 5000 medical students. Student *t*-test and ANOVA were used to document mean differences among different groups. Linear and logistic regression models were used to find predictors of publication and number of publications.

**Results:**

A total of 636 students participated in the survey with a women to men ratio of 1.1. Women medical students report significantly higher knowledge (*t*(634) = 2.47, p = 0.013), personal (*t*(634) = 3.31, p = 0.001), and total barriers scores than men (*t*(634) = 3.02, p = 0.003). Moreover, compared to men, women were less likely to find same-sex mentorship (*t*(634) = 3.18, p = 0.001) or receive credited authorship (*t*(634) = 2.12, p = 0.011). Overall, women medical students were more likely to perceive that their gender (*t*(634) = 3.58, p < 0.001) and people’s perception of their gender (*t*(634) = 4.25, p < 0.001) are barriers to their career advancement. Binary logistic regression demonstrated that gender is a significant predictor of being able to publish (OR: 1.645; 95%CI: 1.002–2.731), while linear regression demonstrated that gender is a predictor of number of publications (ß: 0.113; 95%CI: 0.063–0.288).

**Conclusion:**

A significant gender disparity exists in terms of both attitudes and overall barriers among Jordanian medical undergraduates which calls for immediate policy changes as to produce successful clinicians and researchers.

**Supplementary Information:**

The online version contains supplementary material available at 10.1186/s12909-022-03822-9.

## Background

Literacy and proficiency in medical research are critical for the development of modern clinicians [1, 2]. This has prompted increasing efforts to incorporate research-oriented curricula in undergraduate medical education [3]. The literature documented a multitude of research barriers across both undergraduate and postgraduate levels including lack of protected research time, lack of financial support, absent mentorship, and lack of research knowledge [4, 5]. However, women face additional barriers in comparison to men medical students; those include lack of same sex mentors and role models, gender discrimination, poor work-life balance, and sexual harassment [6, 7]. These hurdles against women has been cultivated through reinforcing gender norms and sustaining gender stereotypes within institutional policies and employees’ bias [8].

In addition to its impact in fortifying one’s scholarly profile, greater research productivity contributes to a myriad of advantages including but are not limited to faster academic ranking, higher salaries, more institutional support for research, less career hurdles, and overall better career satisfaction [9, 10]. Even in terms of research advantages, a gender gap has been widely documented for women [11, 12]. This gap demotivates women researchers with respect to the advancement of their academic careers thus forcing them to publish less scholarly work, set lower career goals, or just drop out of academia – a phenomenon labeled as the “leaky pipeline”.

Research investigating the attitude towards and barriers to research among women medical undergraduates is scarce among Middle Eastern schools, and nonexistent in Jordanian medical schools. Given the advantages of conducing medical research, it is imperative to investigate the barriers which prevent medical undergraduates from engaging in scholarly activities and contribute to the greater body of the literature, especially those within developing nations. Moreover, considering that women within academia are an underrepresented group, examining gender disparity in academic achievement is of vital importance. Therefore, this study primarily aims to identify the attitudes and barriers of Jordanian medical undergraduates towards medical research, as well as finding out the predictors of academic achievement within such a group. The secondary aim of our study is to explore gender disparity within medical research practices.

## Materials and methods

### Setting, sampling and design

We conducted a cross-sectional study exploring research barriers on all Jordanian medical students in their clinical years enrolled between October and November 2021. Jordan has six medical schools harboring more than 5000 medical students in the clinical years only. Students are expected to complete three years of basic sciences followed by three years of clinical training to fully attain the graduation requirements. All of these medical schools have recently adopted research curriculums primarily administered during the clinical years of the six-year program (i.e., 4th, 5th, and 6th years). Among all Jordanian medical schools, students are expected to draft, present, and defend a research manuscript as a perquisite for their graduation with a Doctor of Medicine (M.D.) degree.

The study utilized an online, self-administered questionnaire formulated on Google Forms. The questionnaire was disseminated using social media (e.g., Facebook and WhatsApp) to official groups of targeted medical students at the University of Jordan, Jordan University of Science and Technology, the Hashemite University, Mutah University, Yarmouk University, and Zarqa University. Convenience and snowball sampling were used to reach required sample size. Included were participants that gave an informed consent, completed at least 80% of the questionnaire, and are in their clinical years. Students from the basic sciences years (i.e., 1st, 2nd, and 3rd years) were excluded as they are not subjected to any research activities or courses. This study was approved by the University of Jordan Institutional Review Board and followed the institutional and/or national research committee’s ethical standards and the principles of the World Medical Association’s Declaration of Helsinki.

### Instrument development

The questionnaire utilized for data collection was developed after an extensive literature review. The content validity was further reinforced by two senior faculty experts with significant research experience. The questionnaire is comprised of 4 domains including (1) demographics, (2) research background, (3) attitudes to research, and (4) barriers to research. The fourth and final domain is further sub-divided into three subdomains including knowledge barriers, organizational barriers and miscellaneous barriers. The final questionnaire harbors 43-items (excluding demographics), of which the attitude and barriers domains were presented as 5-point Likert scales.

A pilot test on 30 participants, excluded from the final analysis, was conducted to measure response time, internal consistency of the attitudes and barriers domains, and ensure appropriate interpretation of the questionnaire’s items. The Cronbach α values for the questionnaire’s domains were 0.821 for the attitudes to research domain, 0.773 for the knowledge barriers domain, 0.845 for the organizational barriers’ domain, and 0.737 for the miscellaneous barriers’ domain. The overall Cronbach α for all barriers domains was 0.867. The average time to questionnaire completion was 5 minutes.

### Sample size

The estimated sample size was calculated using GPower 3.1 and EpiInfo. At a power of 95%, an α margin of error of 5% and an effect size of 30%, a sample of 580 participants was needed to demonstrate statistical differences of appropriate power.

### Statistical analysis

Data were analyzed using SPSS version 23. For items utilizing 5-point Likert scales, disagreement responses were grouped together, while agreement responses were grouped together for ease in reporting. Moreover, average 5-point Likert scales were reported as means ± standard deviations. Mean differences of every item within the questionnaire were compared between various categorical groups using the student’s *t*-test and ANOVA. Subsequently, total attitudes and barriers scores were determined by calculating the average mean of all items constituting said domain and compared between categories using the aforementioned statistical tests. The highest mean score is 5 and the lowest is 1, where higher scores indicate more positive attitudes or higher perceptions of barriers. Confidence in leading a research project was measured using an 11-point scale ranging from 0 to 10, which was then categorized into 0–3 (low confidence), 4–7 (moderate confidence), and 8–10 (high confidence). A binary logistic regression model and a linear regression model were computed to explore predictors of being able to publish a research and higher number of publications, respectively.

Multiple correction for multiple t-tests was conducted using the Holm-Bonferroni sequential method, while ANOVA testing was corrected for using the Bonferroni post-hoc method. Correction for regression analysis was not conducted as it was an exploratory analysis of controlled predictors. Also, the conservative nature of correction reduces type I errors on the expense of increasing type II errors. All statistical tests are conducted with a 95% confidence interval and a 5% error margin. A p-value of less than 0.05 is considered statistically significant.

## Results

### Sociodemographic characteristics

Our study recruited a total of 636 participants with a mean age of 22.03 ± 1.36 years. Of the participating cohort, 52.2% were women while 47.8% were men. When stratified by clinical year, 4th year, 5th year, and 6th year students comprised 35.5%, 31.6%, and 32.9% of the total sample, respectively. About 58.4% of sample had ‘very good’ GPA, 26.3% had ‘excellent’ GPA, while 15.3% had ‘good or below’ GPA.

### Research background

More than half of the studied sample were currently working on a research project (54.7%). A total of 313 participants had research training outside their medical school curriculum (49.2%). Overall, 102 students were able to publish a research manuscript in a peer reviewed journal (16.0%), of which, only 18 participants (2.8%) were able to publish as a first author. Mean publications across the entire sample is 0.23 ± 0.77 ranging from 0 to 7. Nonetheless, 52.5% of the sample were willing to pursue a career in academia after graduation while more than 438 students were at least moderately confident in leading a research project (68.9%). The most popular study designs among medical students were cross-sectional studies (40.3%), retrospective cohorts (17.3%), and case reports (14.8%). Table 1 demonstrates the sociodemographic and research characteristics of included participants.

**Table 1:** Characteristics of recruited participants (n = 636)

Variable(s)		n (%)
Gender		
	Men medical students	332 (52.2)
	Women medical students	304 (47.8)
Year of study		
	4th	226 (35.5)
	5th	201 (31.6)
	6th	209 (32.9)
Academic standing*		
	Excellent	116 (26.1)
	Very Good	369 (58.0)
	Good	91 (14.3)
	Satisfactory	6 (0.9)
Currently conducting research		
	Yes	348 (54.7)
	No	288 (45.3)
Number of publications in a peer reviewed journal		
	0	555 (87.3)
	1	45 (7.1)
	2	22 (3.4)
	3 +	14 (2.2)
Previous research training		
	Yes	313 (49.2)
	No	323 (50.8)
Common study designs/types		
	Case report	94 (14.8)
	Retrospective cohort	110 (17.3)
	Cross-sectional	256 (40.3)
	Prospective cohort	25 (3.9)
	Basic science	49 (7.7)
	Randomized Controlled Trial	20 (3.1)
	Systematic reviews	76 (11.9)
	Meta analysis	70 (11.0)
	Case control	4 (0.6)

*Academic standing is based on GPA values out of 4.0. Excellent (GPA ≥ 3.65), Very Good (3.6 > GPA > 3.0), Good (3.0 > GPA > 2.5), Satisfactory (GPA < 2.5)

### Attitudes towards research

The overall mean attitudes score across the studied sample was 43.45 ± 6.95. The overall attitudes of all participating medical students towards the value, benefits, and implications of research were generally positive for all 11 items (Refer to Table 2). Additional details are provided in this file [See Supplementary Tables 1, Additional File 1]. Women were more appreciative of the effect of research on developing sound reasoning (*t*(634) = 2.02, p = 0.044), its implications in residency training and acceptance (*t*(634) = 2.36, p = 0.018), and perceive greater personal gratification from publishing an article (*t*(634) = 2.13, p = 0.033). Post-correction, difference in total attitudes scores between men and women were statistically insignificant (corrected p = 0.094). Table 3 shows the differences between total scores for each tested subscale between men and women.

**Table 2:** Attitudes and perceived barriers among Jordanian medical students stratified by gender

Item	Total (n = 636) Mean ± SD	Women (n = 332) Mean ± SD	Men (n = 304) Mean ± SD	p-value*
**Attitudes**				
Mentorship is important in completing high quality research/scholarly activity	4.54 ± 0.74	4.60 ± 0.72	4.50 ± 0.75	0.109
Research/scholarly activity promotes critical thinking and sound reasoning	4.30 ± 0.86	4.35 ± 0.82	4.22 ± 0.91	**0.044**
Completing research/scholarly activity during residency is important for obtaining a job in a desirable department/location	4.30 ± 0.93	4.33 ± 0.90	4.22 ± 0.95	0.121
I consider research/scholarly activity to be an important part of my residency training	4.02 ± 1.05	4.11 ± 1.04	3.91 ± 1.06	**0.018**
I consider research/scholarly activity during residency is important for obtaining fellowship	4.20 ± 0.95	4.20 ± 0.96	4.20 ± 0.93	0.818
I consider research/scholarly activity during residency is important for achieving an advanced degree	4.15 ± 0.97	4.20 ± 1.00	4.12 ± 0.95	0.598
I would pursue research/scholarly activity even if it were not a mandatory component of residency	3.61 ± 1.30	3.70 ± 1.32	3.54 ± 1.30	0.123
The research/scholarly activity expectations of my department are a source of stress for me	3.53 ± 1.14	3.60 ± 1.11	3.50 ± 1.16	0.142
Research/scholarly activity (e.g., publishing an article) grants me personal gratification	3.94 ± 1.04	4.03 ± 0.97	3.85 ± 1.10	**0.033**
I consider research/scholarly activity as tools that facilitate providing better health services and increase patient care	4.25 ± 0.90	4.31 ± 0.90	4.20 ± 0.92	0.106
Research/scholarly activity should be MANDATORY for medical students and/or residents	3.70 ± 1.24	3.78 ± 1.22	3.60 ± 1.25	0.070
**Knowledge barriers**				
I lack experience in creating a research/scholarly activity proposal/methodology	3.53 ± 1.26	3.65 ± 1.26	3.41 ± 1.24	**0.016**
I lack experience and training in medical writing/manuscript creation	3.60 ± 1.19	3.68 ± 1.19	3.51 ± 1.18	0.066
I have Inadequate linguistic skills (inadequate English) to use academic resources	2.13 ± 1.25	2.15 ± 1.26	2.12 ± 1.24	0.698
I am unable to identify areas of research or formulate research questions	2.87 ± 1.22	2.93 ± 1.27	2.80 ± 1.17	0.177
I am unfamiliar with statistical principles	3.32 ± 1.27	3.46 ± 1.25	3.16 ± 1.27	**0.003**
**Organizational barriers**				
There is lack of protected time for resident research/scholarly activity	4.01 ± 0.89	4.06 ± 0.88	3.96 ± 0.91	0.117
The clinical workload is too high (i.e., interferes with research/scholarly activity time)	4.12 ± 0.90	4.21 ± 0.83	4.03 ± 0.97	**0.013**
There are too many educational activities (e.g., exams and clinical rotations)	4.15 ± 0.92	4.26 ± 0.89	4.04 ± 0.95	**0.002**
There is a lack of funding for research/scholarly activity in my department	4.12 ± 0.91	4.14 ± 0.90	4.09 ± 0.93	0.500
There is a lack of laboratories and other facilities in my department	3.89 ± 1.02	3.96 ± 1.04	3.83 ± 0.99	0.096
There is a lack of faculty members experienced in conducting research	3.41 ± 1.22	3.43 ± 1.24	3.38 ± 1.20	0.593
There is a lack of statistical support	3.84 ± 1.03	3.91 ± 0.98	3.75 ± 1.07	**0.048**
There is lack of ‘‘credited authorship’’ when I participate in research projects	3.46 ± 1.04	3.56 ± 1.05	3.35 ± 1.02	**0.011**
There is difficulty in obtaining data/data collection or inability to recruit participants	3.54 ± 1.09	3.54 ± 1.09	3.54 ± 1.10	0.980
There is a lack of available research/scholarly activity in my department	3.48 ± 1.09	3.51 ± 1.11	3.46 ± 1.08	0.578
Research/ scholarly activity is not perceived to be important by my program/ department	2.94 ± 1.18	2.85 ± 1.17	3.05 ± 1.18	**0.033**
There is difficulty obtaining ethical approval (IRB)	2.99 ± 1.04	2.99 ± 1.07	2.99 ± 1.01	0.997
My program doesn’t have a research/scholarly activity curriculum	2.68 ± 1.21	2.61 ± 1.24	2.75 ± 1.16	0.148
There is a lack of input from research supervisors	3.55 ± 1.07	3.64 ± 1.09	3.45 ± 1.05	**0.029**
There is a lack of cooperation from the authorities of health centers and health staff	3.59 ± 1.04	3.65 ± 1.07	3.52 ± 1.01	0.125
**Misc. barriers**				
The research/scholarly activity projects available are of low quality	3.28 ± 1.07	3.22 ± 1.11	3.34 ± 1.01	0.161
I find it hard to publish projects after completion	3.48 ± 0.96	3.56 ± 0.96	3.39 ± 0.95	**0.024**
I lack encouragement from a mentor	3.61 ± 1.15	3.71 ± 1.13	3.51 ± 1.18	**0.034**
There is difficulty in finding same-gender research mentors	2.61 ± 1.20	2.75 ± 1.24	2.45 ± 1.13	**0.001**
I do not have a personal interest in research/ scholarly activity	2.65 ± 1.44	2.66 ± 1.42	2.65 ± 1.45	0.921
I’m afraid from sexual harassment in research environments	1.81 ± 1.14	2.00 ± 1.18	1.16 ± 1.06	**< 0.001**
Research/scholarly activities are boring	2.87 ± 1.36	2.83 ± 1.33	2.91 ± 1.40	0.478
**Gender perceptions**				
My gender is currently a barrier to my career aspirations/advancement	1.77 ± 1.13	1.92 ± 1.15	1.61 ± 1.07	**< 0.001**
People’s attitudes about my gender are currently a barrier to my career aspirations/advancement	1.89 ± 1.22	2.09 ± 1.26	1.68 ± 1.15	**< 0.001**

*Mean differences between women and men responses to each item were examined by the independent sample *t*-test

### Knowledge research barriers

Among the studied sample, the average knowledge barriers score was 15.45 ± 15.1. Participants have identified hurdles in manuscript writing (58.8%), methodology formulation (57.4%), and lack of familiarity with statistics (46.9%) as their major personal barriers to conducting or publishing a research manuscript. However, the majority of the sample believed that they are able to identify research questions (39.3%) and have adequate linguistic skills to conduct research (67.9%). In terms of gender disparity, women were more likely to report lack of familiarity with statistical principles (*t*(634) = 2.98, p = 0.003) and inability to formulate a comprehensive methodology (*t*(634) = 2.41, p = 0.016) compared to men. Overall, women had a significantly higher knowledge barriers score than men (*t*(634) = 2.48, p = 0.013).

### Organizational barriers

Mean organizational barriers score across the sample was 53.83 ± 8.93. In terms of gender disparity with regards to organizational barriers, women were more likely to perceive higher clinical workloads (*t*(634) = 2.49, p = 0.013) and educational activities (e.g., exams) (*t*(634) = 3.04, p = 0.002) to interfere with research productivity. Moreover, women were less likely to receive statistical support (*t*(634) = 1.98, p = 0.048), credited authorship (*t*(634) = 2.56, p = 0.011), and input from their supervisors (*t*(634) = 2.19, p = 0.029). Nonetheless, mean difference of total organizational barriers score was not statistically significant (*t*(634) = 1.60, p = 0.110).

### Miscellaneous barriers

Miscellaneous barriers were composed of items expressing personal concerns towards scholarly activity as barriers to conducting or completing research. The mean miscellaneous barriers score for the entire sample was 24.01 ± 6.11. Women medical students had significantly higher personal concerns score than their men counterparts (*t*(634) = 3.31, p = 0.001). Also, women were less likely to find a same-gendered mentor (*t*(634) = 3.18, p = 0.001) or find encouragement from mentors (*t*(634) = 2.12, p = 0.034). Additionally, women were more likely to be afraid from sexual harassment within research environment (*t*(634) = 4.24, p < 0.001) and were more likely to perceive that their gender (*t*(634) = 3.58, p < 0.001) and people’s perception of their gender (*t*(634) = 4.25, p < 0.001) are barriers to their career advancement.

### Factors associated with total scores

Significantly more positive attitudes were observed with higher academic standing (F(2,629) = 12.75, p < 0.001), higher confidence in leading a research project (F(2,633) = 10.09, p < 0.001), and previous research training (*t*(634) = 2.48, p = 0.004). Knowledge barriers scores were significantly higher for those with lower academic standing (F(2,629) = 22.95, p < 0.001), lower confidence (F(2,633) = 47.63, p < 0.001), zero publications in peer reviewed journals (*t*(634) = -6.21, p < 0.001), and no previous research training (*t*(634) = -5.786, p < 0.001). Moreover, the overall organizational barriers score was significantly higher for participants that have published research in a peer reviewed journal (*t*(634) = 2.86, p = 0.004).

Higher personal barriers were significantly associated with lower academic standing (F(2,629) = 10.96, p < 0.001), lower confidence in leading a project (F(2,633) = 6.96, p < 0.001), and not working on a project (*t*(634) = -2.46, p = 0.014). Total barriers score among the studied sample was 93.29 ± 15.1. Women had significantly higher total barriers score than men (*t*(634) = 3.02, p = 0.003). Similarly, total barriers score was significantly lower among those with lower academic standing (F(2,629) = 8.57, p < 0.001), zero publications in peer reviewed journals (*t*(634) = -3.03, p = 0.003), lower confidence in leading research projects (F(2,633) = 10.67, p < 0.001), and no other previous research experiences (*t*(634) = -4.19, p < 0.001). For additional details, [See Supplementary Tables 2, Additional File 1].

**Table 3:** Differences between scores between men and women

Score	Men	Women	p-value*
Attitudes score	42.87 ± 7.03	43.97 ± 6.85	0.047**
Knowledge barriers score	14.99 ± 4.46	15.87 ± 4.47	**0.013**
Organizational barriers score	53.24 ± 8.99	54.37 ± 8.85	0.110
Misc. barriers score	23.17 ± 5.95	24.77 ± 6.15	**0.001**
Total barriers score	91.41 ± 14.81	95.02 ± 15.24	**0.003**

*Mean differences across calculated total scores were examined by the independent sample *t*-test

**p-value for mean differences in attitudes score became insignificant post correction with the Holm-Bonferroni method (p = 0.094).

### Multivariate analysis

Binary logistic regression demonstrated that not publishing in a peer reviewed journal was associated with higher knowledge barriers score (OR: 0.925; 95%CI: 0.867–0.990). Men were more likely to report being able to publish in a peer-reviewed journal (OR: 1.645; 95%CI: 1.002–2.731). Similarly, higher confidence (OR: 1.259; 95%CI: 1.118–1.418) was positive predictor of publishing in a peer reviewed journal. Publishing in a peer reviewed journal was also associated with article type including case reports (OR: 2.025; 95%CI: 1.099–3.731), cross sectional studies (OR: 1.965; 95%CI: 1.198–3.225), basic science projects (OR: 2.445; 95%CI: 1.142–5.235), and systematic reviews (OR: 2.900; 95%CI: 1.475–5.772).

On linear regression, men gender was associated with higher number of publications (ß: 0.113; 95%CI: 0.063–0.288). Number of publications was also associated with high confidence (ß: 0.100; 95%CI: 0.005–0.052), and article type including case reports (ß: 0.103; 95%CI: 0.066–0.386), cross sectional studies (ß: 0.149; 95%CI: 0.123–0.350), basic science projects (ß: 0.117; 95%CI: 0.309–0.725) and systematic reviews (ß: 0.150; 95%CI: 0.174–0.547) (refer to Table 4).

**Table 4:** Predictors of publishing and number of publications

	Being able to publish a manuscript in peer reviewed journal (Yes/No)					Number of publications (1–10)				
	**Binary Logistic Regression Model**					**Linear Regression Model**				
	**p-value**	**B**	**Odds Ratio**	**Lower 95% CI for (OR)**	**Upper 95% CI for (OR)**	**p-value**	**B**	**ß**	**Lower 95% CI for (B)**	**Upper 95% CI for (B)**
Men	**0.050**	0.502	1.653	1.000	2.731	**0.002**	0.175	0.113	0.063	0.288
Women (Reference)	**--**	--	--	--	--	**--**	--	--	--	--
GPA	0.594	− 0.181	0.835	0.430	1.622	0.928	0.007	0.003	− 0.139	0.152
Confidence in leading a research project	**0.000**	0.230	1.259	1.118	1.418	**0.019**	0.028	0.100	0.005	0.052
Attitudes score	0.442	− 0.015	0.985	0.947	1.024	0.906	0.001	0.005	− 0.008	0.009
Knowledge barriers score	**0.024**	− 0.078	0.925	0.865	0.990	**0.020**	− 0.019	− 0.107	− 0.034	− 0.003
Organizational barriers score	0.643	− 0.007	0.993	0.962	1.024	0.658	− 0.002	− 0.018	− 0.009	0.005
Miscellaneous barriers score	0.440	0.019	1.019	0.972	1.068	0.835	− 0.001	− 0.009	− 0.012	0.010
Previous research training	**0.037**	− 0.544	0.581	0.348	0.967	**0.040**	− 0.119	− 0.076	− 0.232	− 0.006
Willingness to pursue an academic career	0.172	0.360	1.434	0.854	2.406	0.539	0.036	0.023	− 0.078	0.149
Conducting case reports	**0.024**	0.706	2.025	1.099	3.371	**0.006**	0.226	0.103	0.066	0.386
Conducting retrospective studies	0.616	0.155	1.167	0.637	2.138	0.045	0.152	0.074	0.004	0.300
Conducting cross-sectional studies	**0.007**	0.676	1.965	1.198	3.225	**0.000**	0.236	0.149	0.123	0.350
Conducting prospective studies	0.913	0.059	1.061	0.366	3.074	0.150	0.215	0.054	− 0.078	0.507
Conducting basic science studies (e.g., animal studies)	**0.021**	0.894	2.445	1.142	5.235	**0.000**	0.517	0.177	0.309	0.725
Conducting randomized controlled trials	0.540	0.348	1.146	0.466	4.301	0.475	− 0.114	− 0.026	− 0.428	0.199
Conducting systematic reviews	**0.002**	1.065	2.900	1.457	5.772	**0.000**	0.360	0.150	0.174	0.547
Conducting meta-analyses	0.883	− 0.059	0.943	0.433	2.056	0.228	0.119	0.048	− 0.075	0.314

## Discussion

In our investigation of research barriers among Jordanian medical undergraduates, we demonstrated that women and men report different levels of research barriers which are often perceived more frequently by women. Women had significantly higher knowledge barriers to research compared to men, as they reported subpar statistical knowledge and an inability to formulate concrete methodologies. Moreover, compared to men, women were less likely to find same-sex mentorship, receive credited authorship, or reliably attain encouragement and input from supervisors. Furthermore, women perceive their gender and people’s perception of their gender as hurdles to their career advancement. Nonetheless, undergraduates’ attitudes towards research were generally positive.

Our findings are echoed in the literature as studies conducted in other Arab countries and in the Western world showcased similar results [3, 13–15]. These similarities could be attributed to efforts of policy makers and medical educators all over the globe to increase the exposure of undergraduates to the crucial role of research and its significance. Although some discrepancies across the literature were noted, those may be attributed to the different cultural contexts upon which these studies were conducted. Moreover, inconsistencies in attitudes to certain elements of research could be attributed to the multifaceted nature of attitude itself, as its measurement may not always account for its complex structure pertaining to cognitive, emotional, and behavioral dimensions.

Jordanian medical undergraduates have reported defects in terms of understanding statistics, writing proposals, and designing a rigorous methodology. In fact, their perceived knowledge barriers served as a negative predictor of their ability to publish in peer-reviewed journals or attain higher number of publications. Similar findings were reported in Croatia, United Kingdom, and the Arab world as poor comprehension of basic knowledge in various aspects of scientific theory and statistics were evident [13, 15, 16]. In addition, there was a prominent lack of teaching on proper manuscript drafting and methodology formulation. Our findings might be due to the poor implementation of research policies across Jordanian medical schools, as students involved in extracurricular research training displayed significantly more positive attitudes, lower perception of barriers, and were more likely to publish in peer reviewed journals. The proper implementation and offering of a dedicated research program has proven to improve perceptions of research among medical students [3].

Among our cohort, women medical undergraduates perceived a higher degree of knowledge barriers compared to their men counterparts. A while a knowledge gap in terms of statistical fluency and methodology formulation might be probable, uniformity in biomedical research education might lead us to suggest another explanation. It was demonstrated that women are less likely to present their scientific research with positive words when compared to their men counterparts. Also, men are more likely to self-promote using terms like ‘novel’ or ‘unprecedented’ to describe their scholarly work, thus granting them more publications and leading women to underestimate their abilities [17]. Thus, differences in knowledge barriers might be attributed to variance in self-reported confidence when conducting research tasks.

Jordanian medical schools provide undergraduates with 12 credit hours of research courses in addition to a compulsory graduation project as means to familiarize undergraduates with research essentials. It is, however, critical to note that those implementations could divert from the original intentions if not designed or supervised properly, which might be the case in Jordan. In almost all projects initiated by students, a selected few evolve into publishable manuscripts and a very minimal amount gets published in peer-reviewed journals. This, in return, disallows students from receiving useful feedback on their work upon which they are expected to advance their scholarly competencies.

Among our participants, higher academic standing and perceived self-confidence influenced perceptions of barriers and attitudes towards research. The earlier can be attributed to the association between high GPA and positive attitudes towards the utility of research [3], while the latter only reinforces the role of confidence (i.e., self-efficacy) in facilitating the conduction of successful research, which is well-documented within the literature [18]. Students with higher confidence levels were more likely to publish and have higher number of publications in peer-reviewed journals.

With regards to research design, cross-sectional studies, retrospective cohorts, and case reports were the most prevalent among Jordanian medical undergraduates. Similar patterns were observed among British medical students in which questionnaire-based projects and retrospective chart reviews were the most popular [16]. Furthermore, publishing in peer-reviewed journals and having a high number of publications were associated with article type such as case reports, cross-sectional studies, and systematic reviews. These observations could be attributed to the ease by which these studies are conducted as they require minimal time, effort, and resources in comparison to their longitudinal counterparts.

Our findings demonstrate a deplorable gender disparity where women have significantly portrayed more positive attitudes and higher perceptions of research barriers than their men counterparts. These positive attitudes might be influenced by the increasingly competitive nature of residency spots, both worldwide and within Jordan. It is well documented that research experience boosts the chances of medical graduates getting accepted into the competitive residency programs they desire [19, 20]. This is especially relevant as women might feel the urge to overachieve to be considered equal to their men peers, which prompts a positive demeanor in women towards enrolling in all attainable research opportunities [21]. Furthermore, studies show that women are characterized by higher level of conscientiousness, which has been regarded as a positive predictor of academic achievement [22].

Among our cohort, men were more likely to publish and have a larger sum of publications compared to their women counterparts. Also, women reported being less likely to receive credited authorship. Gender discrimination in authorship has been heavily studied in the developed world and barely any policies were implemented to tackle the issue [23, 24]. It is worthy to mention that such studies are non-existent for the authorship trends in the developing world. Women are often marginalized in peer-reviewed publications, notably in terms of first and senior authorship credit [25]. Moreover, women are less likely to publish in high impact journals, have less citations per publication, and their representation is inversely related with the impact factor of journals [23, 26]. When analyzing the Thesis Awards Committee at the Yale School of Medicine, it was observed that journal reviewers are more likely to reject manuscripts that are conducted by the opposite gender, and since 65% of the committee are men, it is expected that women are more likely to experience more rejection of manuscripts [27].

Overall, recruited women medical undergraduates face more barriers than their men peers which prevents them from participating in research and publishing in an ideal manner. Such barriers include knowledge-oriented hurdles which are related to a reported phenomenon where women demonstrate a deficiency in research-specific skills and statistics [14]. Moreover, women identified significantly higher levels of personal concerns, specifically those pertaining to workload and educational activities. Such findings could be explained within the context of our culture, as in most conservative Arab communities, women tend to have more responsibilities towards their household work and families in contrast to men, thus yielding more difficult life-work balance routines and higher personal concerns [28].

We demonstrate that women medical students were significantly less likely to find same-sex mentors or receive any constructive input from supervisors. While mentorship is important academic productivity and career advancement [27], women in academic medicine face a plethora of difficulties in finding mentors irrespective of the mentor’s gender [29]. For example, top-notch men faculty members are less likely to work with women researchers, thus subjecting them to inferior mentoring [30]. Despite the increasing numbers of women physicians in the medical field, the availability of and access to women mentors are still lacking which further impedes women’s career advancement [29].

By deeply exploring the core definition of mentorship, it was demonstrated that the dyadic mentoring model fails to align with women as it is implemented within sexual dynamics grounded on a male socialization model, in which men root for hierarchy that sets the base of the professional interaction between the mentor and mentee [31]. Hence, the model emphasizes on technical and informational conversations over psychosocial issues, and predominates the value of challenge, competition and independence. This model might not necessarily align with some women as they might favor encouragement over challenge, collaboration over independence, and equalizing behavior over hierarchical behavior [29]. Thus, it is vital for women to get adequate access to same-sex mentors in order for the mentorship process to be in line with their beliefs and ideology.

Finally, women are more likely to be afraid from sexual harassment within research environment than men; an observation that is consistent within the literature [32]. It is reported that more than half of women faculty members experienced gender-based discrimination and sexual harassment, although hardly any men reported going through such experiences or acknowledged that such behavior existed in academic medicine. Such stressful experiences are associated with chronic psychological impairment [33]. Among our sample, women were more likely to perceive their gender as a hurdle to the advancement of their career. Due to them being victims of gender inequalities and sufferers of its consequences, women have an increased sensitivity to gender awareness and its associated issues [34, 35].

Our findings are limited by a variety of factors. The study’s cross-sectional design limited our ability to make more rigorous inferences. The closed-ended questions might have missed additional barriers. Although our study measured many variables, a detailed description of other factors such as student debt, research infrastructure, funding, adequacy of opportunities and others at play is needed.

## Conclusion

Our study meticulously delineated the barriers towards medical research faced by Jordanian medical students. A significant gender disparity exists in terms of both attitudes and overall barriers among medical undergraduates. A multitude of barriers can be conquered by implementing programs aimed at polishing the scholarly skill sets of medical students. Additionally, students should be provided with protected research timed, increased availability of research opportunities, and mentors that suit their differences and research preferences.

## Electronic supplementary material

Below is the link to the electronic supplementary material.

Supplementary Material 1

## Data Availability

The datasets used and/or analysed during the current study are available from the corresponding author on reasonable request.
