# Correction: Perceived barriers towards the importance and application of medical research: a source of gender disparity among medical undergraduates

**DOI:** 10.1186/s12909-022-03964-w

**Published:** 2022-12-19

**Authors:** Lina AlQirem, Leen Al-Huneidy, Muhammad Hammouri, Hana Taha, Husam Al-Somadi, Farah Al-Bitar, Razi Kitaneh, Yazan Al-Huneidy, Hussien Al-Somadi, Omar Ashour, Farah Sayed, Dina Mohammed, Raya Abu Tawileh, Abdallah Al-Ani

**Affiliations:** 1 Faculty of medicine, The University of Jordan, Amman, Jordan; 2 Faculty of medicine, The Hashemite University, Zarqa, Jordan; 3 Department of Internal Medicine, Al-Bashir Hospital, Amman, Jordan; 4 Department of Internal Medicine, Al-Shmaisani Hospital, Amman, Jordan; 5 Department of Internal Medicine, Al-Istishari Hospital, Amman, Jordan; 6 Department of Internal Medicine, Arab Medical Center, Amman, Jordan; 7 Office of Scientific Affairs and Research, King Hussein Cancer Center, Amman, Jordan; 8 Department of Pharmacology, Public Health and Clinical Skills, Faculty of Medicine, The Hashemite University, Zarqa, Jordan

**Correction: BMC Med Educ 22, 767 (2022)**

**https://doi.org/10.1186/s12909-022-03822-9**

Following publication of the original article [1], we have been informed that author Hana Taha has been incorrectly affiliated.

The author affiliation has been updated above and the original article has been corrected.
