# Supplementary material for: Perceived barriers towards the importance and application of medical research: a source of gender disparity among medical undergraduates
Source: BMC Med Educ. 2022 Nov 8;22:767. doi: 10.1186/s12909-022-03822-9 (PMC9644503; doi:10.1186/s12909-022-03822-9)
Supplement: Supplementary file 1 — Supplementary Material 1 [file 12909_2022_3822_MOESM1_ESM.docx]

**Perceived barriers towards the importance and application of medical research: a source of gender disparity among medical undergraduates**

**Supplementary Table 1:** Attitudes and perceived barriers among Jordanian medical students

| **Item** | **Disagree***  **n (%)** | **Neutral***  **n (%)** | **Agree***  **n (%)** |
| --- | --- | --- | --- |
| **Attitudes** |  |  |  |
| Mentorship is important in completing high quality research/scholarly activity | 10 (1.6) | 52 (8.2) | 574 (90.3) |
| Research/scholarly activity promotes critical thinking and sound reasoning | 20 (3.1) | 93 (14.6) | 523 (82.2) |
| Completing research/scholarly activity during residency is important for obtaining a job in a desirable department/location | 28 (4.4) | 98 (15.4) | 510 (80.2) |
| I consider research/scholarly activity to be an important part of my residency training | 58 (9.1) | 125 (19.7) | 453 (71.2) |
| I consider research/scholarly activity during residency is important for obtaining fellowship | 36 (5.7) | 94 (14.8) | 406 (79.6) |
| I consider research/scholarly activity during residency is important for achieving an advanced degree | 43 (6.8) | 94 (14.8) | 499 (78.5) |
| I would pursue research/scholarly activity even if it were not a mandatory component of residency | 131 (20.6) | 129 (20.3) | 376 (59.1) |
| The research/scholarly activity expectations of my department are a source of stress for me | 123 (19.3) | 170 (26.7) | 343 (53.9) |
| Research/scholarly activity (e.g., publishing an article) grants me personal gratification | 52 (8.2) | 153 (24.1) | 431 (67.8) |
| I consider research/scholarly activity as tools that facilitate providing better health services and increase patient care | 34 (5.3) | 81 (12.7) | 521 (81.9) |
| Research/scholarly activity should be MANDATORY for medical students and/or residents | 113 (17.8) | 136 (21.4) | 387 (60.8) |
| **Knowledge barriers** |  |  |  |
| I lack experience in creating a research/scholarly activity proposal/methodology | 148 (23.3) | 123 (19.3) | 365 (57.4) |
| I lack experience and training in medical writing/manuscript creation | 118 (18.6) | 144 (22.6) | 374 (58.8) |
| I have Inadequate linguistic skills (inadequate English) to use academic resources | 432 (67.9) | 100 (15.7) | 104 (16.4) |
| I am unable to identify areas of research or formulate research questions | 250 (39.3) | 192 (30.2) | 194 (30.5) |
| I am unfamiliar with statistical principles | 177 (27.8) | 161 (25.3) | 298 (46.9) |
| **Organizational barriers** |  |  |  |
| There is lack of protected time for resident research/scholarly activity | 28 (4.4) | 153 (24.1) | 455 (71.5) |
| The clinical workload is too high (i.e.: interferes with research/scholarly activity time) | 33 (5.2) | 118 (18.6) | 485 (76.3) |
| There are too many educational activities (e.g., exams and clinical rotations) | 37 (5.8) | 108 (17.0) | 491 (77.2) |
| There is a lack of funding for research/scholarly activity in my department | 24 (3.8) | 149 (23.4) | 463 (72.8) |
| There is a lack of laboratories and other facilities in my department | 49 (7.7) | 189 (29.7) | 398 (62.9) |
| There is a lack of faculty members experienced in conducting research | 154 (24.2) | 180 (28.3) | 302 (47.5) |
| There is a lack of statistical support | 54 (8.5) | 199 (31.3) | 383 (60.2) |
| There is lack of ‘‘credited authorship’’ when I participate in research projects | 89 (14.0) | 269 (42.3) | 278 (43.7) |
| There is difficulty in obtaining data/data collection or inability to recruit participants | 112 (17.6) | 182 (28.6) | 342 (53.8) |
| There is a lack of available research/scholarly activity in my department | 112 (17.6) | 216 (34.0) | 308 (48.4) |
| Research/ scholarly activity is not perceived to be important by my program/ department | 229 (36.0) | 208 (32.7) | 199 (31.3) |
| There is difficulty obtaining ethical approval (IRB) | 176 (27.7) | 291 (45.8) | 169 (26.6) |
| My program doesn’t have a research/scholarly activity curriculum | 281 (44.2) | 207 (32.5) | 148 (23.3) |
| There is a lack of input from research supervisors | 96 (15.1) | 214 (33.6) | 326 (51.3) |
| There is a lack of cooperation from the authorities of health centers and health staff | 80 (12.6) | 230 (36.2) | 326 (51.3) |
| **Misc. barriers** |  |  |  |
| The research/scholarly activity projects available are of low quality | 134 (21.1) | 260 (40.9) | 242 (38.1) |
| I find it hard to publish projects after completion | 65 (10.2) | 296 (46.5) | 275 (43.2) |
| I lack encouragement from a mentor | 106 (16.7) | 170 (26.7) | 360 (56.6) |
| There is difficulty in finding same-gender research mentors | 273 (42.9) | 238 (37.4) | 125 (19.7) |
| I do not have a personal interest in research/ scholarly activity | 339 (53.3) | 99 (15.6) | 198 (31.1) |
| I’m afraid from sexual harassment in research environments | 472 (74.2) | 103 (16.2) | 61 (9.6) |
| Research/scholarly activities are boring | 261 (41.0) | 158 (24.8) | 217 (34.1) |
| **Gender perceptions** |  |  |  |
| My gender is currently a barrier to my career aspirations/advancement | 484 (76.1) | 90 (14.2) | 62 (9.7) |
| People’s attitudes about my gender are currently a barrier to my career aspirations/advancement | 455 (71.5) | 104 (16.4) | 77 (12.1) |

**Supplementary table 2:** Factors associated with total attitudes and barriers scores

|  |  | Attitude Score | **p-value**** | Knowledge Barriers Score | **p-value**** | Organizational Barriers Score | **p-value**** | Misc. Barriers Score | **p-value**** | Total Barriers Score | **p-value**** |
| --- | --- | --- | --- | --- | --- | --- | --- | --- | --- | --- | --- |
| Gender |  |  | **0.047** |  | **0.013** |  | 0.110 |  | **0.001** |  | **0.003** |
|  | Male | 42.87 ± 7.03 |  | 14.99 ± 4.46 |  | 53.24 ± 8.99 |  | 23.17 ± 5.95 |  | 91.41 ± 14.81 |  |
|  | Female | 43.97 ± 6.85 |  | 15.87 ± 4.47 |  | 54.37 ± 8.85 |  | 24.77 ± 6.15 |  | 95.02 ± 15.24 |  |
| GPA |  |  | **<0.001** |  | **<0.001** |  | 0.465 |  | **<0.001** |  | **<0.001** |
|  | Excellent | 45.65 ± 6.12 |  | 13.92 ± 4.43 |  | 53.26 ± 9.28 |  | 22.27 ± 5.67 |  | 89.46 ± 15.12 |  |
|  | Very Good | 43.07 ± 6.85 |  | 15.57 ± 4.31 |  | 54.21 ± 8.89 |  | 24.37 ± 6.26 |  | 94.16 ± 14.93 |  |
|  | Good or below | 41.12 ± 7.67 |  | 17.67 ± 4.34 |  | 53.44 ± 8.64 |  | 25.60 ± 5.73 |  | 96.72 ± 15.01 |  |
| Publication status |  |  | 0.167 |  | **<0.001** |  | **0.004** |  | 0.174 |  | **0.003** |
|  | No | 43.28 ± 6.80 |  | 15.92 ± 4.15 |  | 52.91 ± 9.65 |  | 24.15 ± 5.95 |  | 94.08 ± 14.54 |  |
|  | Yes | 44.32 ± 7.69 |  | 13.00 ± 5.30 |  | 54.00 ± 8.78 |  | 23.25 ± 6.84 |  | 89.16 ± 17.41 |  |
| Confidence in leading a project |  |  | **<0.001** |  | **<0.001** |  | 0.556 |  | **0.001** |  | **<0.001** |
|  | High | 46.26 ± 6.48 |  | 12.67 ± 5.24 |  | 53.95 ± 8.97 |  | 22.90 ± 7.94 |  | 89.54 ± 17.40 |  |
|  | Moderate | 43.77 ± 6.44 |  | 15.19 ± 4.00 |  | 53.48 ± 8.86 |  | 23.60 ± 5.63 |  | 92.28 ± 14.20 |  |
|  | Low | 41.34 ± 7.39 |  | 17.45 ± 3.80 |  | 54.34 ± 9.04 |  | 25.28 ± 5.47 |  | 97.08 ± 14.54 |  |
| Year of study |  |  | 0.774 |  | 0.128 |  | 0.144 |  | 0.863 |  | 0.337 |
|  | 4^th^ year | 43.71 ± 6.63 |  | 15.50 ± 4.21 |  | 52.89 ± 8.47 |  | 23.83 ± 5.77 |  | 92.22 ± 13.83 |  |
|  | 5^th^ year | 43.28 ± 6.96 |  | 15.89 ± 4.36 |  | 54.38 ± 8.97 |  | 24.10 ± 6.32 |  | 94.38 ± 15.54 |  |
|  | 6^th^ year | 43.32 ± 7.30 |  | 14.99 ± 4.85 |  | 54.31 ± 9.33 |  | 24.11 ± 6.27 |  | 93.41 ± 16.05 |  |
| Previous research training |  |  | **0.004** |  | **<0.001** |  | **0.004** |  | 0.058 |  | **<0.001** |
|  | No | 42.67 ± 7.06 |  | 16.44 ± 4.50 |  | 54.82 ± 8.97 |  | 24.46 ± 5.87 |  | 95.73 ± 14.94 |  |
|  | Yes | 44.25 ± 6.75 |  | 14.43 ± 4.23 |  | 52.80 ± 8.79 |  | 23.54 ± 6.32 |  | 90.78 ± 14.94 |  |
| Currently working on research |  |  | **<0.001** |  | **<0.001** |  | 0.660 |  | **0.014** |  | **0.004** |
|  | No | 42.32 ± 6.93 |  | 16.54 ± 3.93 |  | 54.00 ± 8.78 |  | 24.66 ± 5.67 |  | 95.20 ± 13.73 |  |
|  | Yes | 44.38 ± 6.84 |  | 14.56 ± 4.71 |  | 53.68 ± 9.06 |  | 23.46 ± 6.40 |  | 91.71 ± 16.04 |  |
